# Neuroimmune and Neuropathic Responses of Spinal Cord and Dorsal Root Ganglia in Middle Age

**DOI:** 10.1371/journal.pone.0134394

**Published:** 2015-08-04

**Authors:** William Galbavy, Martin Kaczocha, Michelino Puopolo, Lixin Liu, Mario J. Rebecchi

**Affiliations:** Department of Anesthesiology, Stony Brook University, Stony Brook, New York, United States of America; Toronto University, CANADA

## Abstract

Prior studies of aging and neuropathic injury have focused on senescent animals compared to young adults, while changes in middle age, particularly in the dorsal root ganglia (DRG), have remained largely unexplored. 14 neuroimmune mRNA markers, previously associated with peripheral nerve injury, were measured in multiplex assays of lumbar spinal cord (LSC), and DRG from young and middle-aged (3, 17 month) naïve rats, or from rats subjected to chronic constriction injury (CCI) of the sciatic nerve (after 7 days), or from aged-matched sham controls. Results showed that CD2, CD3e, CD68, CD45, TNF-α, IL6, CCL2, ATF3 and TGFβ1 mRNA levels were substantially elevated in LSC from naïve middle-aged animals compared to young adults. Similarly, LSC samples from older sham animals showed increased levels of T-cell and microglial/macrophage markers. CCI induced further increases in CCL2, and IL6, and elevated ATF3 mRNA levels in LSC of young and middle-aged adults. Immunofluorescence images of dorsal horn microglia from middle-aged naïve or sham rats were typically hypertrophic with mostly thickened, de-ramified processes, similar to microglia following CCI. Unlike the spinal cord, marker expression profiles in naïve DRG were unchanged across age (except increased ATF3); whereas, levels of GFAP protein, localized to satellite glia, were highly elevated in middle age, but independent of nerve injury. Most neuroimmune markers were elevated in DRG following CCI in young adults, yet middle-aged animals showed little response to injury. No age-related changes in nociception (heat, cold, mechanical) were observed in naïve adults, or at days 3 or 7 post-CCI. The patterns of marker expression and microglial morphologies in healthy middle age are consistent with development of a para-inflammatory state involving microglial activation and T-cell marker elevation in the dorsal horn, and neuronal stress and satellite cell activation in the DRG. These changes, however, did not affect the establishment of neuropathic pain.

## Introduction

Normal healthy aging is associated with neuroimmune changes that have been referred to as “inflammaging”, an elevation of inflammatory tone with age that may contribute to the aging process itself, as well as enhance susceptibility to neurodegeneration [1–5]. As a result, an incipient or para-inflammatory state is thought to develop that predisposes the senescent CNS to deleterious neurotoxic responses following injury or infection or stress. A large body of evidence now supports this idea. For example, multiple inflammatory markers increase with age in various brain regions of healthy rats, mice, and primates [5–7], particularly the pro-inflammatory cytokines interleukin 1β (IL1β), tumor necrosis factor α (TNFα) and interleukin 6 (IL6), as well as microglial activation markers, Cd11b (Ox42, C3A receptor) and MHCII (major histocompatibility complex II), and the astrogliosis marker, glial fibrillary acidic protein (GFAP); moreover, challenging the senescent CNS with lipopolysaccharide (LPS) or with mechanical injury induces exaggerated neuroinflammatory responses, exacerbates decline, and delays functional recovery [6–9]. Furthermore, neuroimmune profiles of healthy aged and diseased brains suggest that early para-inflammatory changes, particularly activation of microglia [10], may contribute to neurodegenerative disorders, such as Alzheimer’s dementia [11, 12] and Parkinson’s disease [13].

In contrast to the extensive work on the aging mammalian brain, relatively few reports have examined inflammatory markers in the aging spinal cord in healthy or nerve-injured subjects. Early work showed some differences in the numbers of Ox42+ (CD11b) microglia in lumbar spinal cord (LSC) from healthy young and middle-aged adults, whereas senescent adults had greater numbers and staining intensities of activated microglia [14]. Similarly, sections of spinal cords and brainstems from healthy senescent rats showed increased CD11b and ED1 (CD68) immunoreactivity in microglia, and GFAP in astrocytes compared to young adults [15]. Many of these CD68-positive microglia were hypertrophic with short stout processes, many were localized to the white matter, and these were found at higher levels in senescent animals with severe sensorimotor deficits. While it has been reported that sciatic nerve injury increases the numbers of CD11b-positive microglia in both young and middle-aged LSC, this increase was attenuated in senescent animals [14, 16]. In canine spinal cord, increased numbers of Iba1-positive microglia with “activated” morphology have been found in lumbar and cervical cords of older (10–12 years) compared to young adults (1–2 years)[17]. Taken together, these studies demonstrate age-related changes in spinal cord microglia and astrocytes that are consistent with inflammaging and that could lead to exaggerated responses and/or to delayed recovery following nerve injury. Indeed, increased sensitivities to noxious heat [18–20], and increased mechanical allodynia [21] and hyperalgesia [20] have been found in older neuropathic animals. Contrary to these reports, however, reduced mechanical allodynia and decreased ongoing pain have been reported in older rats following spinal nerve ligation [22]. Nonetheless, these evoked response differences were modest, and their interpretation could be complicated by age-related changes in sensory thresholds. On the other hand, substantial delays in pain resolution have been consistently reported in senescent animals following nerve injury [23–25].

Peripheral nerve injury provokes a rapid innate immune response in the DRG and spinal cords of young adult animals [26–29]. Levels of cytokines, including IL1β, TNFα, IL6, IFNγ, and chemokines, such as CCL2, as well as markers of activated microglia, macrophages, astrocytes and T-cells increase after injury. Many, for example TNFα [30], CCL2 [31, 32] and IFNγ [33, 34], are critical for full development of neuropathic pain, and introducing each is sufficient to mimic important aspects of pain development. Anti-inflammatory cytokines, such as IL10 and IL4, also increase, and help transform macrophages and microglia from reactive to reparative and immunosuppressive phenotypes that drive development [28, 35], and possibly resolution of neuropathic pain. This body of work has led to the prevalent view that damage to primary sensory neurons entrains glial dependent, and innate immune reactions that develop into neuropathic pain which may resolve or remain persistent. A comprehensive examination of these responses in aging spinal cord and DRG following peripheral nerve injury, however, is lacking and few studies have included animals of middle age, a time when increased prevalence and duration of chronic pain reaches a plateau in humans [36–40]. Here we report on neuroimmune changes in LSC and DRG of middle-aged animals compared to young adults and their responses to neuropathic injury.

## Materials and Methods

### Chronic Constriction Injury

Fisher 344 rats of 3–5 and 15–19 months of age were obtained from the NIA colony and housed at the Division of Laboratory Animal Resources in a 12 h light/dark cycle. All work conformed to the National Institutes of Health Guidelines for the Care and Use of Laboratory Animals and were approved by the Stony Brook University Institutional Animal Care and Use Committee. Animals were double-housed before surgery and single-housed after surgery, receiving food and water ad libitum. The chronic constriction injury, or CCI model of neuropathic pain, originally described by Bennett and Xie [41], was performed with slight modification to improve reproducibility of the injury. Briefly, each animal received an intraperitoneal injection of ketamine/xylazine (75 mg/kg and 5 mg/kg, respectively). The hind leg was shaved and surgically prepared with 70% ethanol and Triadine, and the anesthetized animal placed prone on a sterile towel over a heating pad. The temperature was monitored rectally. A skin incision was made mid-thigh with a medium curved scalpel and then surgical scissors were used to expose the sciatic nerve. Approximately 1 cm of the main trunk proximal to the trifurcation was isolated and 4 strands of 4–0 chromic gut were tied around the nerve ~ 1 mm apart under magnification. In a modification of the original method, a strand of 2–0 prolene was placed between the nerve and the gut against which the suture was tightened. The prolene strand was then removed. This prevented over tightening and reduced occurrence of subsequent motor paralysis. Sham surgeries involved exposing the sciatic nerve without applying chromic gut. The muscle layers were then re-opposed and sutured, and surgical staples were used to close the skin. None of the animals included in our experiments exhibited signs of significant motor paralysis.

### Behavioral Measurements

All animals were habituated to the apparatuses and then subjected to evoked behavioral responses at baseline and at 3 and 7 days, while a parallel group was measured every week thereafter up to 35 days post-surgery. The Hargreaves test was used to assess thermal hyperalgesia. Briefly, plexiglass enclosures were set atop a plexiglass platform and a 200 mW, 535 nm diode laser was mounted in an adjustable stand placed beneath the plexiglass platform, and was used as the heat stimulus. The latency time to hind paw lift during heating of the plantar surface was recorded. Five recordings were obtained on each hind paw with at least 2 min rest between measurements. The maximum time for exposure to the diode laser source was 25 s to avoid any possible tissue injury. For mechanical threshold measurements, the animal enclosures rested on a screen with mesh of 0.5 cm spacing. Following acclimation, an electronic von Frey Anesthesiometer (IITC Life Sciences) was applied with increasing static pressure to the plantar surface of the hind paw until the animals lifted the hind paw. The number of grams of force applied by the probe to induce withdrawal was recorded. Five recordings were obtained on each hind paw with at least 2 min between measurements. Cold allodynia was measured as the cumulative attention response time to the affected limb caused by evaporative cooling using a volume (0.1ml) of acetone administered to the dorsal aspect of the hind paw.

### Perfusion, Fixation and Tissue Harvesting

At the appropriate times, rats were euthanized and transcardially perfused with heparinized saline-buffered with 5 mM H_2_NaPO_4_ to pH 7. Ipsilateral and contralateral LSC hemi-sections, and L4 and L5 DRG were removed and immediately frozen on dry ice. In some animals, transcardial perfusion with heparinized saline was followed by 4% formaldehyde freshly prepared in phosphate buffered saline (PBS). These *in situ* fixed, dissected tissues specimens were then post-fixed for 1 h in 4% formaldehyde in PBS at room temperature before being transferred to a solution of 30% sucrose in PBS for overnight incubation at 4°C. Fixed tissues were then embedded in OCT medium (HistoPrep, Fisher Chemical), and frozen on dry ice. All samples were stored at -8°C.

### QuantiGene 2.0 Multiplex Assay

Total RNAs were extracted from LSC hemi-sections and lumbar DRG (L4-L5) using Qiazol extraction, and further purified with RNeasy spin columns following the manufacturer’s directions. Briefly, frozen tissues were placed on ice, Qiazol lysis reagent (Qiagen) was added immediately along with three or six 2.3 mm silica/zirconia beads (DRG and LSC, respectively), and homogenized in a BioSpec mini bead beater for 1.5 min and allowed to stand on ice for 5 min. Chloroform was added to comprise 1/5 of the total volume, and samples were mixed vigorously for 2 min and allowed to settle for 2 min before being centrifuged at 12,000 X g for 15 minutes at 4° C. The upper aqueous phase was saved, mixed 1:1 with 70% ethanol, and subjected to RNeasy spin column purification (Qiagen). Final concentrations and 230/260/280 ratios were determined by nanodrop absorbancy using an Eppendorf BioSpectrometer. Quantigene 2.0 Plex Assays were performed on the purified RNA’s. 10 plex and 18 plex probe sets were designed based on previously identified key inflammatory and nerve injury related genes [33]. Assays were first shown to be linear with respect to RNA concentrations (50 To 500 ng/well) with final assays conducted using 500 ng/well of RNA in triplicate. Two housekeeping genes were assayed in each set (Hprt1 and Pplb), which did not change significantly with age. The plates were read on a BioPlex 200 (Bio Rad). After subtracting background, the results were normalized to the geomean of the two control genes for each well.

### Immunoblotting

Proteins were extracted from the frozen samples with ice cold lysis buffer prepared with complete EDTA-free protease inhibitor cocktail tablets (Roche Pharmaceuticals), 20 mM Tris, 150 mM NaCl, 2.5 mM Na_4_P_2_O_7_, 1% Nonidet P40, 0.1% SDS and 1 mM each of EDTA, NaF, PMSF, Na_3_VO_4_, and dithiothrietol. Three or six beads (DRG and SC, respectively) of 2.3 mm diameter Zircona/Silica (Biospec) were added to the samples that were then homogenized in a Biospec Mini Bead Beater for one min and placed on ice (if necessary, samples were homogenized again if visible particulates were still present after sitting for 3 minutes on ice). Samples were centrifuged at 4°C at 13,000 × g for 15 min and the supernatant fluids were saved. Total protein concentrations were determined using Bio Rad Protein Assay Dye Reagent Concentrate. Concentrated Laemmli buffer was added to the extracts and samples were heated at 85°C for 1 min and stored frozen at -20^°^C. Equal amounts of total protein were loaded onto 10% polyacrylamide gels and were subjected to SDS-PAGE in a minigel apparatus (BioRad) and transferred using 0.05% SDS, 10% methanol in Transfer Buffer to PVDF membrane at 22 V for 2 h in a Semi-Dry Blot apparatus (BioRad). Membranes were blocked overnight with 5% non-fat dry milk in Tris-buffered saline (TBS) at 4°C and probed with rabbit polyclonal Iba1 antibody (Wako, 019–19741) at a dilution of 1:500 with 5% BSA in 0.05% Tween-20 in Tris-buffered saline (TBS-T) or mouse monoclonal GFAP antibody (UC Davis/NIH NeuroMab, 75–240) at 1:6000 for 3 h. Internal controls were performed with GAPDH antibody (Sigma G8795) incubated at 1:8000 with 5% non-fat dry milk in TBS for 3 h. After primary antibody incubation, membranes were washed 3 times with TBS + 0.05% Tween-20 (TBS-T) for 10 min each wash. Secondary antibodies (goat-anti-mouse or goat anti-rabbit IgG linked to HRP) were diluted 1:6000 (Invitrogen Zymax goat anti-mouse, 81–6520) or 1:3000 (Santa Cruz goat anti-rabbit, L1911) with 5% non-fat milk in TBS and incubated with the washed membranes for 2 h shaking at room temperature. Membranes were then washed 3 times again with TBS-T as described above and antibody binding was detected with ECL plus reagent (GE) on a C-Digit western blot scanner model 3600 (LI-COR). Bands were analyzed using the C-Digit Image studio software according to the formula: [(band pixel intensity/area)–(background pixel intensity/area)] / [(housekeeping band pixel intensity/area)–(background pixel intensity/area)].

### Immunofluorescence, Imaging, and 3D Renderings

Indirect immunofluorescence was used to assess morphologies of microglia and astrocytes in LSC and to localize GFAP expression in satellite glia in DRG. Fixed OCT embedded frozen tissues were cut into 25 μm thick transverse sections with a cryostat (Leica) and collected onto Superfrost Plus microscope slides. Dry sections were immediately stored up to several days at −20°C. Before the addition of antibody, the sections were permeabilized and blocked with 10% goat serum (GS) in TBS with 0.6% Nonidet P40 for 1 h at room temperature. Specimens were then probed with primary antibody overnight in 10% GS in TBS with 0.3% Nonidet P40 at 4°C. Iba1 rabbit polyclonal and GFAP mouse monoclonal antibodies were diluted 1:400 in TBS containing 10% goat serum. Slides were washed 3 times in TBS-T for 10 min each, and incubated with fluorescently labeled goat anti-rabbit IgG (Alexa Fluor488, Molecular Probes) or goat anti-mouse IgG (Alexa Fluor594 Molecular Probes) diluted 1:500 in 10% goat serum and 1% rat serum with 0.3% Nonidet P40 in TBS for 2 h at room temperature. Wash steps were repeated, and the slides were dipped once in deionized water and thoroughly drained. A drop of mounting fluid, Prolong Gold Antifade Reagent with DAPI (Molecular Probes) was placed on each section and coverslips were mounted. The tissues were then imaged on a laser scanning confocal microscope (Olympus Fluoview-1000). LSC Iba1 images were obtained with a 20X dry objective for merges with dorsal horn transmitted light images. Z stacks of 25 images were captured with a 40X 1.4 NA lens oil objective in increments of 1.25 μm, comprising 31.25 μm. LSC and DRG GFAP images were obtained with a 60X 1.4NA objective lens, with Z stack optical sections at intervals of 0.75 μm through the 25μm thick specimens. All images were exported as TIFF files to Image J for processing. A Zeiss LSM 510 META-NLO Two-Photon Laser Scanning Microscope was also used with a 100X 1.4 NA objective lens to capture high-resolution Z-axis image stacks that were reconstructed into rotatable 3D images using the Zeiss LSM Image Browser.

### Morphological Analysis

Fractal Analysis software (FracLac ImageJ plugin) was utilized for morphological analysis as originally described in Smith et al, J. Neurosci Methods, 1996. Z stack images of spinal cord dorsal horns were despeckled, thresholded and binarized before being subjected to fractal analysis. The program settings used were 12 grid positions, 100 grid calibers or box sizes, minimum sampling pixel size of 3, maximum size of sampling elements 2% of image, with horizontal slide X = 10 pixels. Data were plotted as the log N (box counts) and log e (box scale/image scale). D_B_, the fractal dimension, was determined from the slope of this plot, which is a measure of complexity related to the numbers of processes and their branching; where D_B_ = -lim[log N_i_*e/log e], N_i_ = i^th^ box counting grid and e = box scale/image scale.

Iba1 stained microglia in young and middle-aged spinal cord dorsal horn z stacks were categorized according to morphologic phenotype (n = 3 animals per group, 2 LSC dorsal horn sections per LSC spaced at least 50 μm apart). Additionally total microglia were counted (n = 3 animals per group, 4 dorsal horn sections per LSC spaced at least 50 μm apart). Two categories were defined to organize the total counts of microglia: phenotype 1 (P1) characteristics: small cell body, numerous long processes with extensive process arborization. P1 also includes cells that are highly ramified, with a “bushy” morphology, where processes are not as long, but had numerous branches per process. Phenotype 2 (P2) comprised any Iba1+ microglia that did not fit the description of P1. Their characteristics were more variable and included hypertrophic cell body, shorter stouter processes with less extensive arborization, as well as microglia that were elongated with a polarized distribution of processes. Microglia crossing the boundaries of the optical fields were not counted.

### Statistics

Significance of behavioral differences between sham and CCI injured animals and across ages at a single time point were determined by Two-Way ANOVA followed by Tukey’s multiple comparisons post-test. For comparisons to baseline behavioral measurements, Repeated Measures Two-way ANOVA was employed with Sidak’s multiple comparisons test. Two-way ANOVA followed by Tukey’s multiple comparisons post-test was also used to examine Quantigene expression differences across injury (sham vs CCI) and age (young vs middle-aged). For age-related naive differences, student t-tests were performed with multiple comparisons FDR adjustment of 5% [42]. Significance of differences in relative immunoblotting intensities across age or injury was determined by student t-test with Bonferroni correction. χ2 test was used to assess the significance of differences from expected phenotype (P1, P2) values between young and middle-aged groups. All levels of adjusted p values for significance were p<0.05.

## Results

### Age-Related Changes in the Lumbar Spinal Cord and DRG

#### I. Expression of Neuroinflammatory Markers in Naïve LSC and DRG

To determine whether healthy middle-aged lumbar spinal cord (LSC) and dorsal root ganglia (DRG) show signs of inflammaging that could influence their responses to or recovery from nerve injury, expression levels of multiple neuroinflammatory mRNA markers were measured in the healthy young (2–3 month) and middle-aged (17 month) animals using a custom Quantigene 2.0 Multiplex assay. This assay was designed to quantify mRNA levels of cytokines and chemokines, known to initiate, maintain or modulate central neuroinflammatory pathways including IL1β, TNFα, IL6, IL2, IFNγ, IL10, IL4, and CCL2, as well as mRNA corresponding to cell-type markers CD2, CD3e (T-cells), CD68 (macrophages and microglia), GFAP (astrocytes/satellite cells), CD45 (T cells and macrophages), and ATF3 (neuronal stress). Levels were measured in samples of LSC and L4-L5 DRG and normalized to the geomeans of two housekeeping genes that did not vary significantly with age, HPRT1 and PPIB. LSC GFAP mRNA levels were beyond the upper bounds of the linear range of our assay, whereas IL2 and IL4 levels were too low (</ = 2 x assay background) in both LSC and DRG samples, and so were excluded.

**Fig 1:**
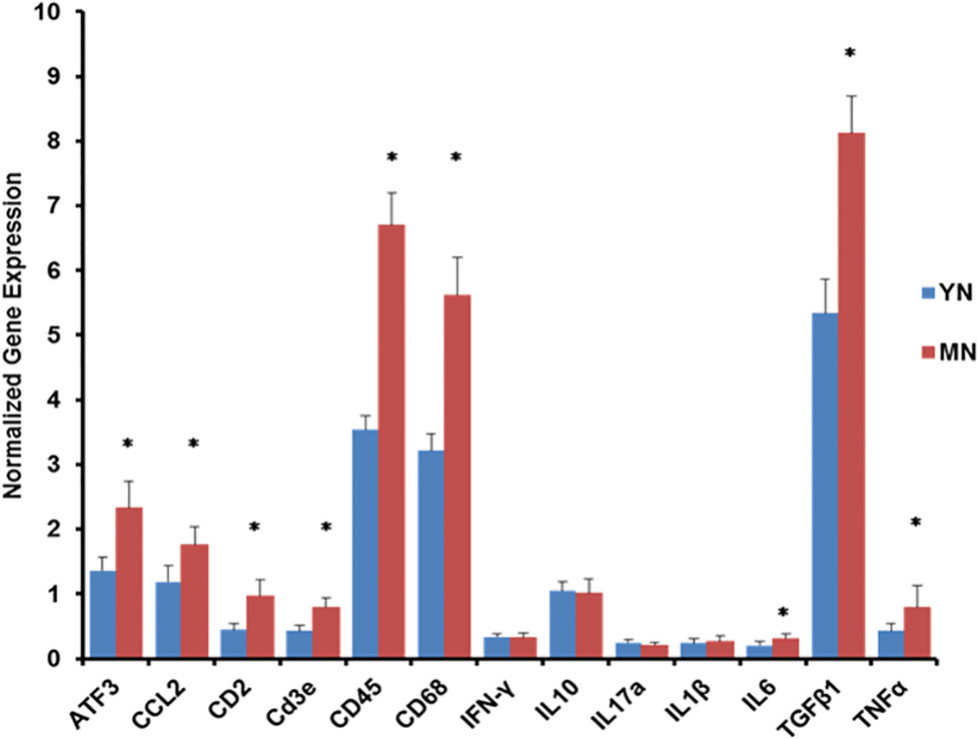
Neuroimmune gene expression profile in lumbar spinal cords of naïve young and middle-aged rats. Expression levels were normalized to the geomean of Hprt1 and Pplb expression and the ratios multiplied by 100. Results are presented as means +/- SD. Significance of differences between YN v MN * p < 0.0015.

Many of these markers were significantly elevated in the LSC of middle-aged naïve adults (Fig 1), particularly T cell and microglial/macrophage markers, ATF3 (stress), as well as TNFα, a pro-inflammatory cytokine, and CCL2, a chemokine implicated in microglial activation and establishment of neuropathic pain [31]. Up-modulation of TGFβ1, a powerful anti-inflammatory immunomodulator [43], was also observed. In DRG, however, only ATF3 was elevated in middle age (Table 1).

**Table 1:** Gene expression Profile of Naïve Young and Middle-Aged DRG.

Gene	YN Mean	SD	n	MN Mean	SD	n	MN/YN	p value
***ATF3***	**5.61**	**0.75**	**5**	**8.21**	**0.83**	**5**	**1.46**	**0.00041**
CCL2	15.3	3.02	12	15.2	1.63	11	0.99	0.46567
CD2	1.97	1.05	12	1.79	0.62	11	0.91	0.30773
CD3E	1.08	0.71	5	0.94	0.23	5	0.87	0.34092
CD45	5.53	1.32	5	5.63	0.45	5	1.02	0.43584
CD68	5.69	1.75	12	4.89	0.87	11	0.86	0.09305
GFAP	14.4	10.7	5	16.0	6.24	5	1.11	0.39410
IFNγ	0.81	0.59	12	0.69	0.36	11	0.85	0.28904
IL10	3.30	2.73	5	1.87	1.38	5	0.57	0.16347
IL17a	1.41	0.71	7	1.27	0.47	6	0.90	0.34050
IL1β	0.32	0.10	12	0.32	0.06	11	1.00	0.46804
IL6	0.98	0.54	12	0.91	0.33	11	0.93	0.36072
TGFβ1	6.31	1.48	5	5.79	0.62	5	0.92	0.24515
TNFα	2.84	1.31	7	2.78	1.11	6	0.98	0.46832

Results are expressed as mean +/-SD and numbers of animals assayed per group are indicated. Bolded/italicized genes indicate significant differences. Further statistical results and corrections for multiple comparisons are in Additional File 1. YN = young naïve, MN = middle-aged naïve.

#### II. Microglial Morphology

**Fig 2:**
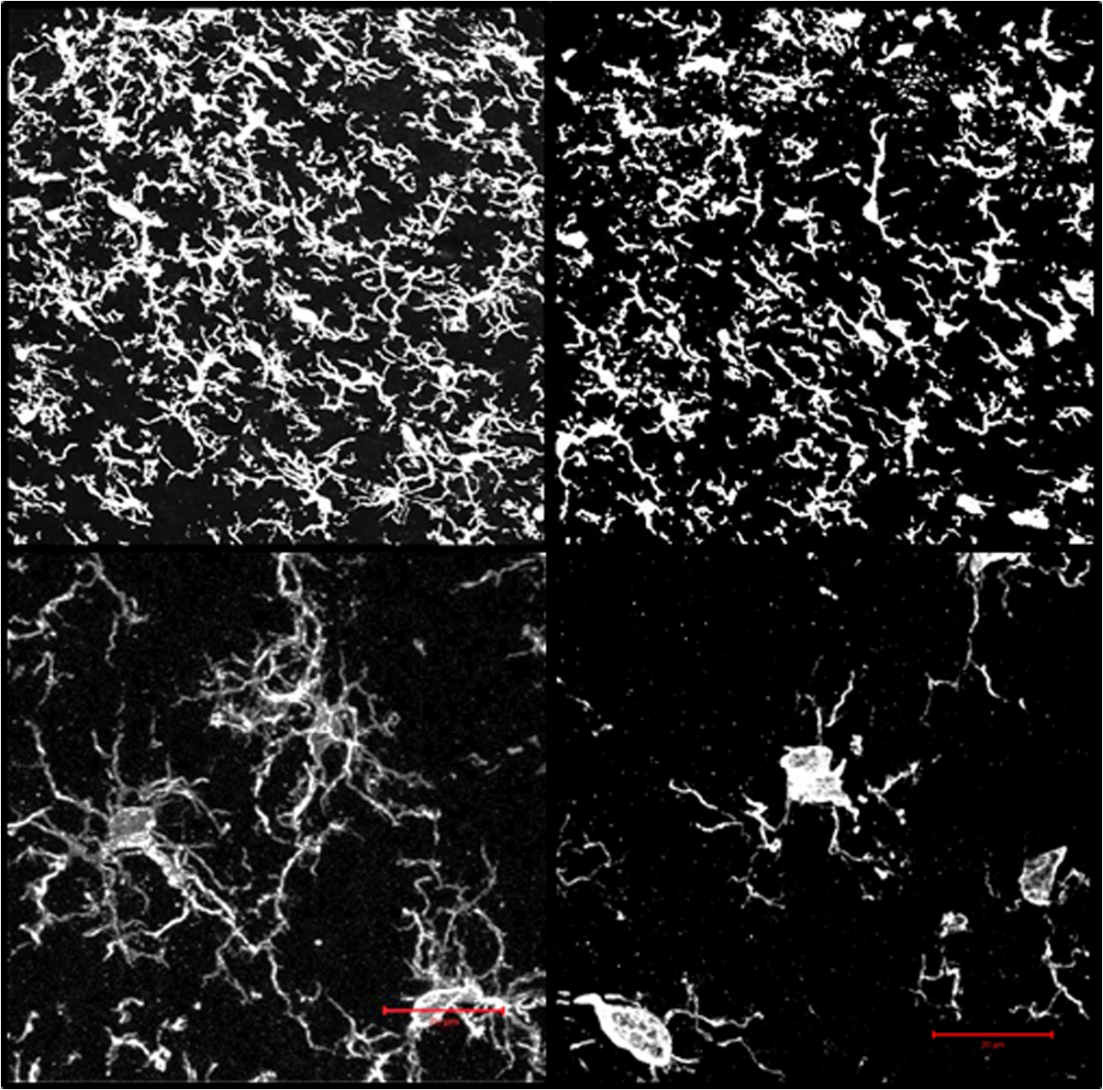
Iba1 immunofluorescence images of the ipsilateral dorsal horns from naïve young (YN) and middle-aged (MN) rats rendered in 3D. Upper Panels: individual optical sections (1.25 μm intervals through 25 μm thick sections) were acquired with a LSCM and 40x 1.4 NA objective lens and then recombined and rendered as three-dimensional images. Lower Panels: Two-photon immunofluorescence 3D renderings of Iba1 positive dorsal horn microglia from young and middle-aged naïve (YN and MN). Image stacks (0.44 μm optical sections through 25 μm thick specimens) were obtained using a two-photon laser scanning confocal microscope and 100 X 1.4 NA objective lens. Each stack set was recombined to create the 3D rendering. A single plane of the 3D image is shown for each. Scale bar = 25 μm. Rotatable 3D images are also available in S1 and S2 Files.

To assess whether changes in microglial morphology, that may reflect a pro-inflammatory phenotype, could arise in the spinal cord dorsal horn by 17 months of age, LSC from naive young and middle-aged rats were fixed *in situ*, frozen, sectioned and stained with antibody to Iba1 (ionized calcium-binding adapter molecule 1, also known as AIF1), a specific marker of microglia and macrophage activation [44]. The differences were clear. Young naïve dorsal horn microglia had compact cell bodies with highly branched processes; whereas middle-aged naïve microglia were typified by hypertrophic cell bodies, and shortened and thickened processes with relatively few branches (Fig 2, upper panels). The stark differences between the two age groups are even more apparent at higher resolution (Fig 2, lower panels—two-photon images stacks with 3-D reconstructions are displayed; see S1 and S2 Files for rotatable images.

**Fig 3:**
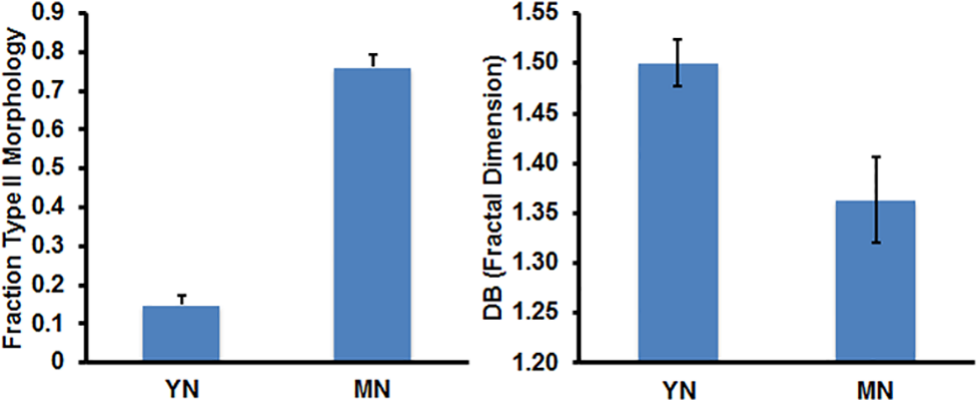
Analyses of confocal images of Iba1+ cells in sections of lumbar spinal cord dorsal horns from young or middle-aged naïve rats (Left) Fraction of Iba1+ lumbar microglia exhibiting P2 morphology (see METHODS for description); data are presented as mean +/- SD, n = 3 rats per age group, and 2 independent spinal cord sections per animal; significance of difference in proportions of P2 morphology in YN and MN is p < 0.0001. (Right) D_B_ is the fractal dimension, a measure of complexity related to the numbers of microglial processes and branching; where D_B_ = -lim[logN_ε_/log_ε_], N_i_ = i^th^ box counting grid and ϵ = box scale/image scale. The non-overlapping 95% confidence intervals of the means of six D_B_ determinations for n = 3 per age group and 2 independent LSC sections per animal.

To quantify age-related morphological changes, two categories were devised to classify naïve dorsal horn microglia: phenotype 1 and 2 (P1, P2). P1 is characterized by small cell bodies with multiple, long processes, and extensive arborizations per process, characteristics that have been associated with “quiescent” microglia. P2 comprise any Iba1+ microglia that did not fit the P1 description. P2 phenotypes generally showed fewer, shorter processes and significant cytoplasmic hypertrophy characteristics typifying primed, activated and alternatively activated microglia, but also included highly elongated cells, as well. Although such a binary classification system simplifies the considerably more diverse functional phenotypes [6, 28, 45], it was highly convenient for morphologic analysis. Dorsal horn microglia of middle-aged naïve LSC were comprised of 76.2 +/- 3.5% of P2 type, whereas young naïve LSC had only 14.9 +/- 2.4% P2 type (n = 3 naïve rats per age group; 2 independent dorsal horn fields per animal) (Fig 3, Left). These marked changes suggest that age contributes to development of distinctive LSC microglial morphologies in otherwise healthy animals. On the other hand, total dorsal horn microglial cell counts did not differ significantly between age groups: YN = 455 and MN = 481 total Iba1+ cells (n = 3 naïve rats per age group; 4 independent dorsal horn fields per animal).

Fractal analysis, an independent approach to assess morphologic complexity [46], including that of microglia [47], was used to help quantify the age-related differences in microglial process branching in the dorsal horn LSC images. The fractal analysis results were plotted as the log N (box counts) and log e (box scale/image scale), which are variables related to complexity where the slope, D_B_ = -lim[log N_i_*e/log e], N_i_ = i^th^ box counting grid and e = box scale/image scale. Higher D_B_ (fractal dimension) is consistent with increased numbers of processes and their branching. Compiled results in Fig 3 (Right) show that the log ratio D_B_, was higher in images of young adult microglia (~10%) compared to those in middle age. Multiple determinations of D_B_ were reproducible with non-overlapping 95% confidence intervals. These data support the differential partitioning of morphologies between young and middle-aged microglia as discussed above.

### Age-Related Changes in Neuroimmune Marker Expression and Glial Morphology following Nerve Injury

#### I. CCI Induced Expression of Neuroinflammatory Markers in LSC

To address possible age-related differences in the central neuroimmune response to nerve injury, expression levels of neuroinflammatory mRNA markers were determined using the same QuantiGene 2.0 Multiplex assay as described above. Levels were measured in samples of LSC (hemi-sections ipsilateral (IPSL) to nerve injury or sham surgery) and L4-L5 DRG (CCI or sham surgery). CCI increased ATF3, CCL2, and IL6 mRNA levels in IPSL LSC at post-surgery day 7 compared to age-matched sham controls in both young and middle-aged animals (Table 2); in young CCI LSC, their up-modulation was > 3 fold. These changes are consistent with increased neuronal stress, resulting in up-modulation of cytokines and chemokines, including CCL2, which has been shown to activate and recruit microglia to the affected side [48].

**Table 2:** Gene Expression Profile of Young and Middle-Aged Sham and CCI LSC.

**Gene**	**YS Mean**	**SD**	**n**	**YCCI Mean**	**SD**	**n**	**Fold YCCI/YS**	**p value**
***ATF3***	**1.60**	**0.05**	**4**	**8.79**	**1.68**	**5**	**5.49**	**0.0001**
***CCL2***	**1.30**	**0.30**	**9**	**4.53**	**1.04**	**9**	**3.49**	**0.0001**
CD2	0.57	0.09	9	0.41	0.18	9	0.72	0.2834
CD3E	0.45	0.08	4	0.46	0.11	5	1.02	0.9991
***CD45***	**3.65**	**0.63**	**4**	**7.18**	**1.68**	**5**	**1.97**	**0.0033**
CD68	3.62	0.52	9	5.23	0.83	9	1.44	0.1862
IFN-γ	0.36	0.09	4	0.50	0.19	5	1.39	0.9441
IL10	1.31	0.26	4	1.38	0.69	5	1.05	0.9927
IL17a	0.38	0.12	4	0.25	0.13	5	0.66	0.9999
IL1β	0.23	0.05	9	0.31	0.13	9	1.35	0.2120
***IL6***	**0.26**	**0.07**	**9**	**0.80**	**0.21**	**9**	**3.08**	**0.0001**
TGFβ1*	5.94	0.52	4	8.03	2.03	5	1.35	0.0883
TNFα	0.38	0.11	4	0.45	0.05	5	1.18	0.6654
**Gene**	**MS Mean**	**SD**	**n**	**MCCI Mean**	**SD**	**n**	**Fold MCCI/MS**	**p value**
***ATF3***	**2.28**	**0.34**	**5**	**5.98**	**0.44**	**5**	**2.62**	**0.0001**
***CCL2***	**1.93**	**0.37**	**9**	**3.42**	**1.00**	**10**	**1.77**	**0.0010**
CD2	0.83	0.19	9	0.74	0.24	10	0.89	0.7400
CD3E	0.84	0.06	5	0.88	0.17	5	1.05	0.9470
CD45	7.05	1.05	5	8.31	1.22	5	1.18	0.3987
***CD68***	**5.81**	**0.87**	**9**	**8.49**	**2.93**	**10**	**1.46**	**0.0068**
IFN-γ	0.41	0.06	5	0.44	0.10	5	1.07	0.9747
IL10	1.41	0.28	5	1.42	0.17	5	1.01	0.9999
IL17a	0.30	0.07	5	0.26	0.05	5	0.87	0.9135
IL1β	0.30	0.06	9	0.26	0.09	10	0.87	0.8157
***IL6***	**0.30**	**0.09**	**9**	**0.68**	**0.20**	**10**	**2.27**	**0.0001**
TGFβ1	8.39	0.58	5	9.65	0.90	5	1.15	0.3811
TNFα	0.46	0.13	5	0.55	0.03	5	1.20	0.4756

LSC ipsilateral hemisections were removed on day 7. Expression levels in LSC samples were normalized to the geomean of Hprt1 and Pplb expression and the ratios multiplied by 100 and presented as the mean ratios +/- SD. Two-way ANOVA results are shown with contrasts and associated p values. Numbers of animals (n) assayed per group are shown. Bolded/italicized genes indicate specific contrast differences p < 0.05

* indicates difference trending to significance. YS = young sham, YCCI = young CCI, MS = middle-aged sham, MCCI = middle-aged CCI.

CD45 increased significantly in young CCI LSC (~2 fold), and TGFβ1 levels trended upwards (1.35 fold), although the differences between YS and YCCI in TGFβ1 levels did not attain statistical significance (p = 0.0883). CD68, a specific microglial/macrophage marker, appeared to be elevated 1.4 fold, but also did not reach significance in young adults. Middle-aged CCI animals showed >2 fold increases in ATF3 and IL6 in LSC compared to age-matched sham controls, as well as increases in CCL2 and CD68 that were statistically significant (Table 2). IL1β, TNFα and IFNγ, however, were not significantly elevated in young or older CCI animals compared to sham controls.

Comparisons of sham controls across age revealed elevated expression of the T cell markers CD2 and CD3e, microglial/macrophage marker CD68, and leukocyte marker CD45, in middle-aged LSC (Table 3). Similarly, increased levels of CD2, CD3e and CD68 were observed in middle-aged CCI compared to young CCI LSC. ATF3 and CCL2 were not as highly induced by CCI in the middle-aged animals. Taken together, these data suggest the presence of T cells and novel microglial states in middle age LSC.

**Table 3:** Lumbar Spinal Cord Gene Expression Comparing Sham and CCI: Effects of Age.

Gene	Fold MS/YS	p value	Gene	Fold MCCI/YCCI	p value
ATF3	1.42	0.6892	***ATF3***	**0.68**	**0.0011**
CCL2	1.48	0.3138	***CCL2***	**0.76**	**0.0178**
***CD2***	**1.47**	**0.0332**	***CD2***	**1.80**	**0.0031**
***CD3E***	**1.87**	**0.0007**	***CD3E***	**1.91**	**0.0002**
***CD45***	**1.93**	**0.0045**	CD45	1.16	0.4927
***CD68***	**1.60**	**0.0403**	***CD68***	**1.62**	**0.0009**
IFN-γ	1.14	0.9982	IFN-γ	0.88	0.9999
IL10	1.08	0.9802	IL10	1.03	0.9993
IL17a	0.79	0.9983	IL17a	1.04	0.9640
IL1β	1.30	0.3799	IL1β	0.84	0.5967
IL6	1.15	0.9387	IL6	0.85	0.3855
***TGFβ1***	**1.41**	**0.0385**	TGFβ1	1.20	0.1894
TNFα	1.21	0.5129	TNFα	1.22	0.3342

LSC ipsilateral hemisections were removed on day 7. Expression levels in lumbar spinal cord were normalized to the geomean of Hprt1 and Pplb expression and the ratios multiplied by 100 and presented as the mean ratios +/- SD. Two-way ANOVA results are shown with contrasts and associated p values. Numbers of animals (n) assayed per group are shown. Bolded/italicized genes indicate specific contrast differences p < 0.05

* indicates difference trending to significance. Abbreviations are as in Table 2.

#### II. Neuroinflammatory Gene Expression in DRG following CCI

Of the 14 genes profiled, 10 were differentially expressed after nerve injury in young L4/L5 DRG compared to age-matched shams (Table 4). In young animals, IL6, CD68, IL1β, and CCL2 expression increased markedly along with TNFα, CD2, ATF3, IL17a, GFAP and IFNγ. These results agree with previous studies of IL6, ATF3, and IL1β and TNFα expression in DRG following CCI [49–51]. Increases in T-cell and macrophage markers are also consistent with infiltration of young adult DRG after nerve injury [52]. In middle age, however, only CCL2 and IL6 were significantly elevated following CCI (Table 5).

**Table 4:** Gene Expression Profile of Young Sham and CCI DRG at Day 7.

Gene	YS Mean	SD	n	YCCI Mean	SD	n	Fold YCCI/YS	p value
***ATF3***	**11.9**	**0.34**	**5**	**99.9**	**63.3**	**5**	**8.39**	**0.0304**
***CCL2***	**16.7**	**1.43**	**10**	**29.8**	**3.81**	**9**	**1.78**	**0.0001**
***CD2***	**0.74**	**0.34**	**10**	**1.49**	**0.87**	**9**	**2.01**	**0.0220**
CD3E	0.58	0.56	5	0.47	0.18	5	0.81	0.9598
CD45	4.76	0.72	5	7.07	1.22	5	1.48	0.1899
***CD68***	**3.55**	**0.94**	**10**	**9.24**	**3.51**	**9**	**2.60**	**0.0001**
***GFAP***	**14.4**	**8.04**	**5**	**30.7**	**9.27**	**5**	**2.13**	**0.0441**
**IFN-γ**	**0.20**	**0.15**	**10**	**0.47**	**0.30**	**9**	**2.35**	**0.0448**
IL10	1.27	1.70	5	0.76	0.29	5	0.60	0.7778
***IL17a***	**0.25**	**0.28**	**5**	**1.36**	**0.93**	**4**	**5.44**	**0.0320**
***IL1β***	**0.23**	**0.06**	**10**	**0.51**	**0.16**	**9**	**2.22**	**0.0001**
***IL6***	**0.50**	**0.28**	**10**	**6.23**	**2.79**	**9**	**12.5**	**0.0001**
TGFβ1*	5.66	0.95	5	7.71	1.28	5	1.36	0.1226
***TNFα***	**0.71**	**0.52**	**5**	**3.44**	**1.71**	**4**	**4.84**	**0.0039**

**Table 5:** Gene Expression Profile of Middle-Aged Sham and CCI DRG at Day 7.

Gene	MS Mean	SD	n	MCCI Mean	SD	n	Fold MCCI/MS	p value
ATF3	32.2	31.2	5	69.8	53.7	6	2.17	0.5260
***CCL2***	**17.4**	**2.51**	**9**	**34.7**	**7.14**	**10**	**1.99**	**0.0001**
CD2	1.19	0.29	9	1.38	0.47	10	1.16	0.8640
CD3E	0.80	0.29	5	0.81	0.30	6	1.01	0.9999
CD45	7.69	2.44	5	6.89	1.95	6	0.90	0.8712
CD68	5.59	1.32	9	6.63	1.56	10	1.19	0.6886
GFAP	18.5	6.71	5	25.0	10.5	6	1.35	0.6285
IFN-γ	0.30	0.13	9	0.36	0.24	10	1.20	0.9351
IL10	0.54	0.24	5	0.57	0.33	6	1.06	0.9999
IL17a	0.82	0.24	4	1.17	0.39	4	1.43	0.7730
IL1β	0.28	0.10	9	0.36	0.08	10	1.29	0.3928
***IL6***	**0.73**	**0.26**	**9**	**5.36**	**2.85**	**10**	**7.34**	**0.0001**
TGFβ1	8.05	1.88	5	7.34	1.25	6	0.91	0.8293
TNFα	1.59	0.28	4	2.34	0.70	4	1.47	0.6843

Two-way ANOVA results (normalized as described in Table 1) of dorsal root ganglia expression levels are shown with contrasts and associated p values and numbers of animals assayed per group. Bolded/italicized genes indicate specific contrast differences p < 0.05

* indicates difference trending to significance.

Abbreviations are as in Table 2.

Comparisons of young and middle-aged sham control DRG suggested that CD45 and TGFβ1 trended higher in older animals (1.61 and 1.42 fold, respectively), but these results did not reach statistical significance (Table 6). In middle-aged CCI compared to young CCI animals, only CD68 and IL1β were significantly different, and slightly depressed (0.72 and 0.71, respectively). Thus, older animals did not produce a robust neuroimmune response in the DRG following nerve injury, like that seen in young adults.

**Table 6:** DRG Gene Expression Comparing Sham and CCI: Effects of Age.

Gene	Fold MS/YS	p value	Gene	Fold MCCI/YCCI	p value
ATF3	2.71	0.8902	ATF3	0.70	0.6927
CCL2	1.04	0.9842	CCL2*	1.16	0.0862
CD2	1.61	0.2815	CD2	0.93	0.9693
CD3E	1.38	0.7717	CD3E	1.72	0.4092
CD45*	1.61	0.0691	CD45	0.97	0.9982
CD68	1.57	0.1547	***CD68***	**0.72**	**0.0412**
GFAP	1.29	0.8798	GFAP	0.82	0.722
IFN-γ	1.50	0.7557	IFN-γ	0.77	0.6489
IL10	0.43	0.5449	IL10	0.75	0.9853
IL17a	3.28	0.4017	IL17a	0.86	0.9526
IL1β	1.22	0.7231	***IL1β***	**0.71**	**0.0209**
IL6	1.46	0.9945	IL6	0.86	0.7814
TGFβ1*	1.42	0.0605	TGFβ1	0.95	0.9681
TNFα	2.24	0.5179	TNFα	0.68	0.3829

Two-way ANOVA results (normalized as described in Table 1) of dorsal root ganglia are shown with contrasts and associated p values and numbers of animals assayed per group. Bolded/italicized genes indicate specific contrast differences p < 0.05

* indicates difference trending to significance.

Abbreviations are as in Table 2.

#### III. Changes in Microglia, Astrocytes, and Satellite Glia following CCI

**Fig 4:**
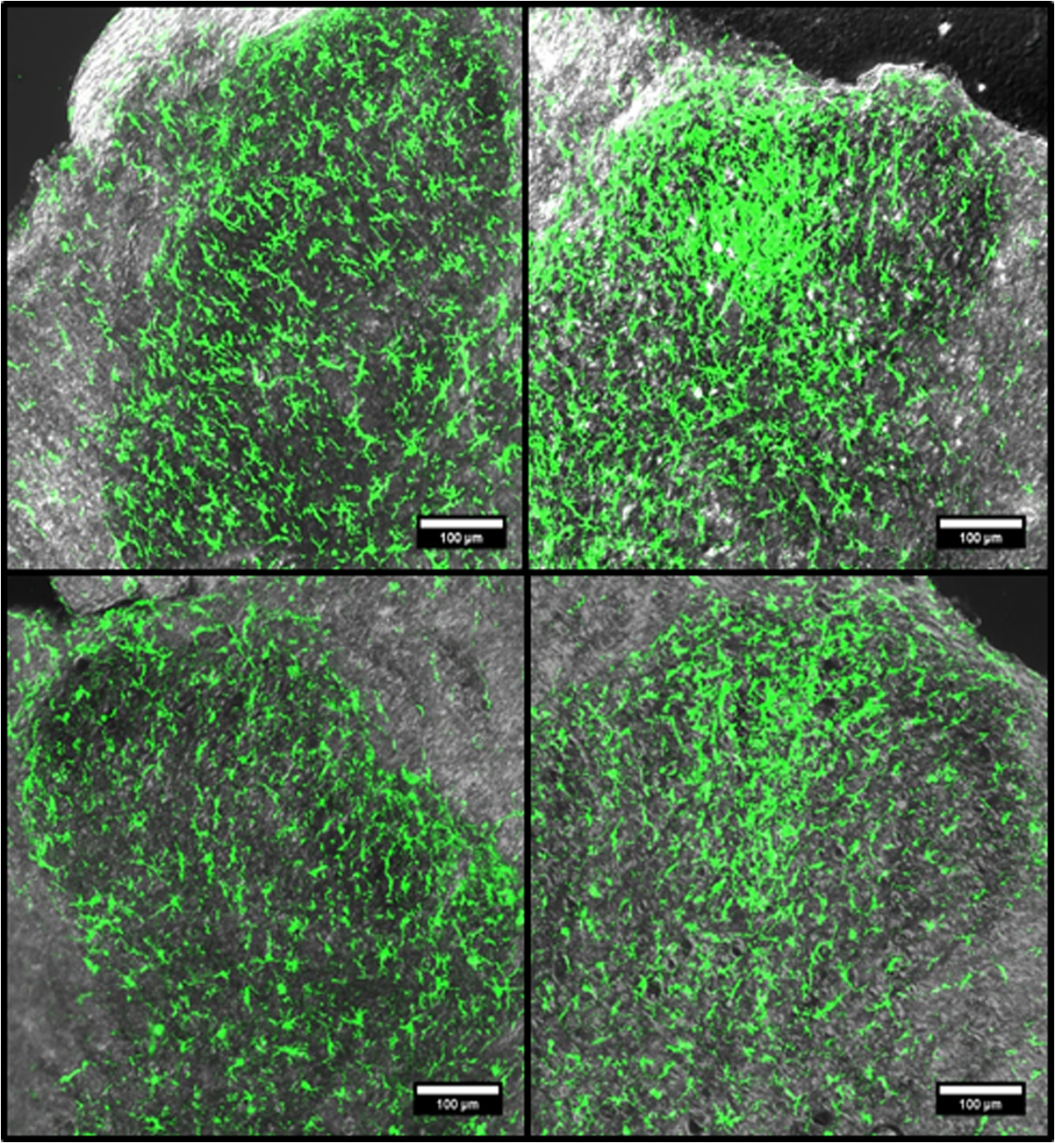
Representative Iba1 immunofluorescence images of young and middle aged of lumbar spinal cord dorsal horns from post-CCI day 7 animals, ipsilateral or contralateral to injury. Immunofluorescence confocal images of the dorsal horns stained with Iba1 antibody were combined with corresponding transmitted light images. Young (YCCI) and middle-aged (MCCI) dorsal horns ipsilateral (IPSL) to injury are compared to the contralateral (CL) sides. Scale bars = 100 μm.

**Fig 5:**
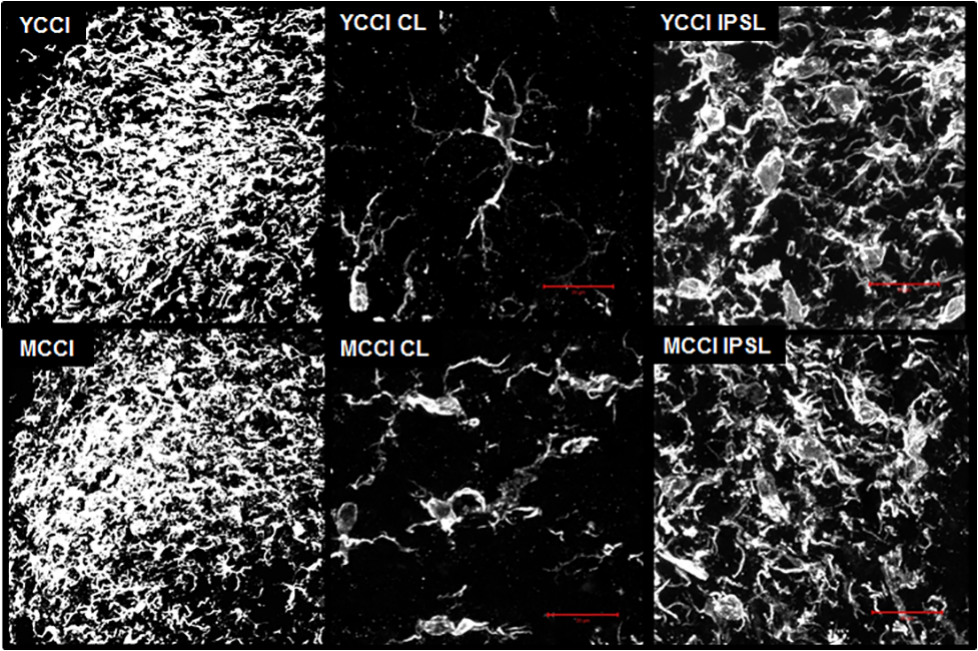
Iba1 immunofluorescence of the ipsilateral dorsal horns from post-CCI day 7 young (YCCI) and middle-aged (MCCI) rats rendered in 3D. Left Panels: optical sections (1.25 μm intervals through 25 μm thick sections) were acquired with a LSCM and 40x 1.4 NA objective lens and then recombined and rendered as three-dimensional images. Middle and Right Panels: image stacks (0.44 μm optical sections through 25 μm thick specimens) were obtained using a two-photon laser scanning confocal microscope and 100 X 1.4 NA objective lens. Each stack set was recombined to create the 3D rendering. A single plane of the 3D image is shown for each. Scale bar = 25 μm.

To assess whether age affected the morphologic, and proliferative or migratory responses of microglia to injury, LSC from CCI animals were fixed *in situ*, frozen, sectioned and stained with Iba1. Microglia, stained with Iba1, accumulated in the LSC IPSL dorsal horns of young and middle-aged animals 7 days post-CCI. Both age groups showed a concentration of highly fluorescent microglia adjacent to the most lateral aspect of the dorsal tract in the superficial dorsal horn and towards the center (Fig 4). High-resolution two-photon image stacks of contralateral (CL) and ipsilateral (IPSL) dorsal horn microglia from CCI animals were obtained. 3D images were reconstructed from these stacks and are displayed in a single view plane (Fig 5; for 3D rotatable images of dorsal horn microglia from animals subjected to CCI, see S3–S6 Files). Both young and middle-aged CCI IPSL microglia exhibited hypertrophic cell bodies and de-ramified processes (Fig 5, right panels) consistent with activated morphologies previously described in nerve injured animals [28]. In the contralateral dorsal horn, MCCI microglia were noticeably different from their young counterparts, with larger cell bodies and shorter, stouter processes with less branching (Fig 5, middle panels). Thus, CL MCCI microglia were distinct from the CL YCCI, but similar to the naïve middle-aged microglia described above.

**Fig 6:**
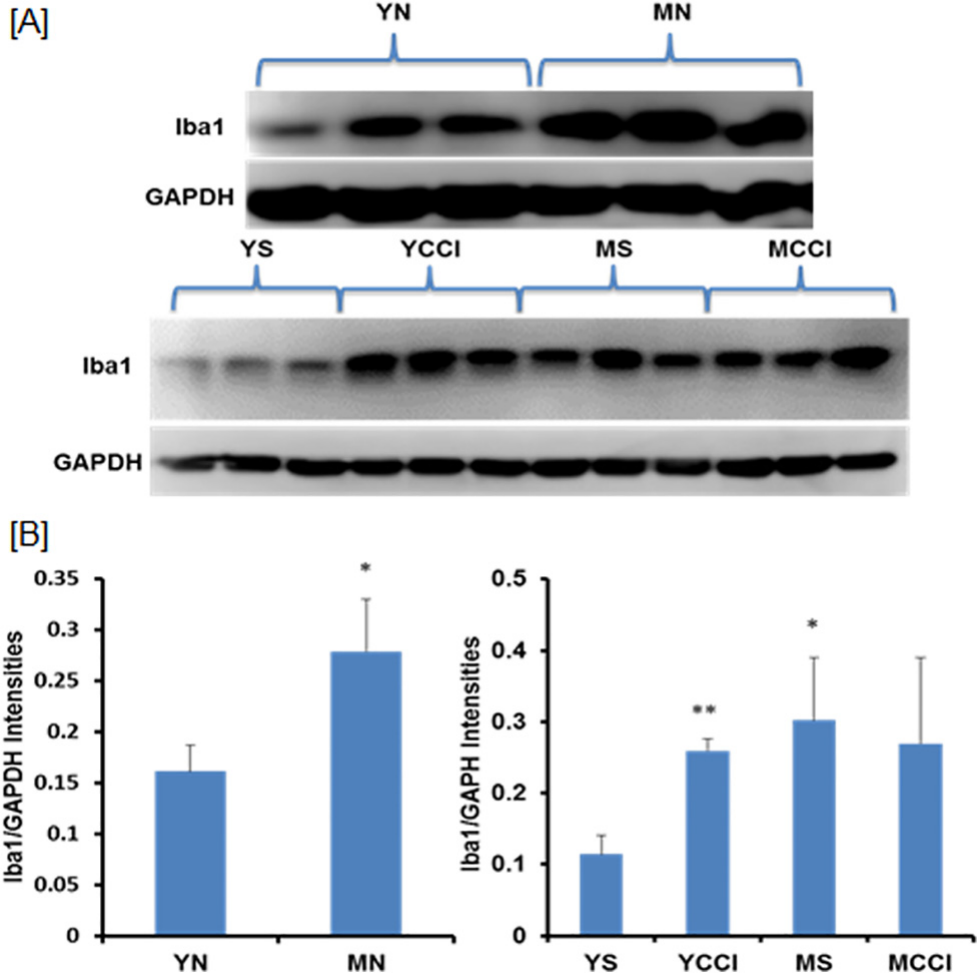
Levels of Iba1 protein expression in young and middle-aged sham and post-CCI day 7 lumbar spinal cords. Results were obtained for each sample (n = 3 per age group). A) immunoblots of lumbar spinal cord hemisection samples from naïve animals (Top), and samples ipsilateral to injury (Bottom), were probed with antibodies against the microglial marker Iba1 and house keeping enzyme GAPDH (loading control). B) Results are expressed as the mean ratios Iba1/GAPDH intensities +/- SD; significance YN v MN (*p = 0.0120), left panel, YS v YCCI (**p = 0.0006), YS v MS (*p = 0.0121) and MS v MCCI (p = 0.3500), right panel.

To evaluate age and injury related microglial activation in the LSC, samples of IPSL LSC from sham and CCI groups were subjected to SDS-PAGE and immunoblotted with an antibody to Iba1. The levels of Iba1 were calculated from the ratios of Iba1/GAPDH immunoblot intensities. CCI induced a >2 fold increase in Iba1 levels in young animals 7 days post CCI compared to age-matched sham controls, while middle-aged CCI animals saw no corresponding increase (Fig 6A and 6B). Expression of Iba1, however, was already ~3 fold higher in the middle-aged sham compared to young sham controls, and Iba1 levels were also significantly increased in middle-aged naïve LSC compared to young naïve LSC (Fig 6A and 6B). These changes were concordant with increased expression of microglia/macrophage marker CD68 and the leukocyte marker, CD45 in LSC from older animals (Fig 1). Thus, LSC expression of Iba1 is already increased in middle-aged adults, consistent with age-related phenotypic changes in microglia in the absence of neuropathic injury.

### GFAP Protein Expression in LSC

**Fig 7:**
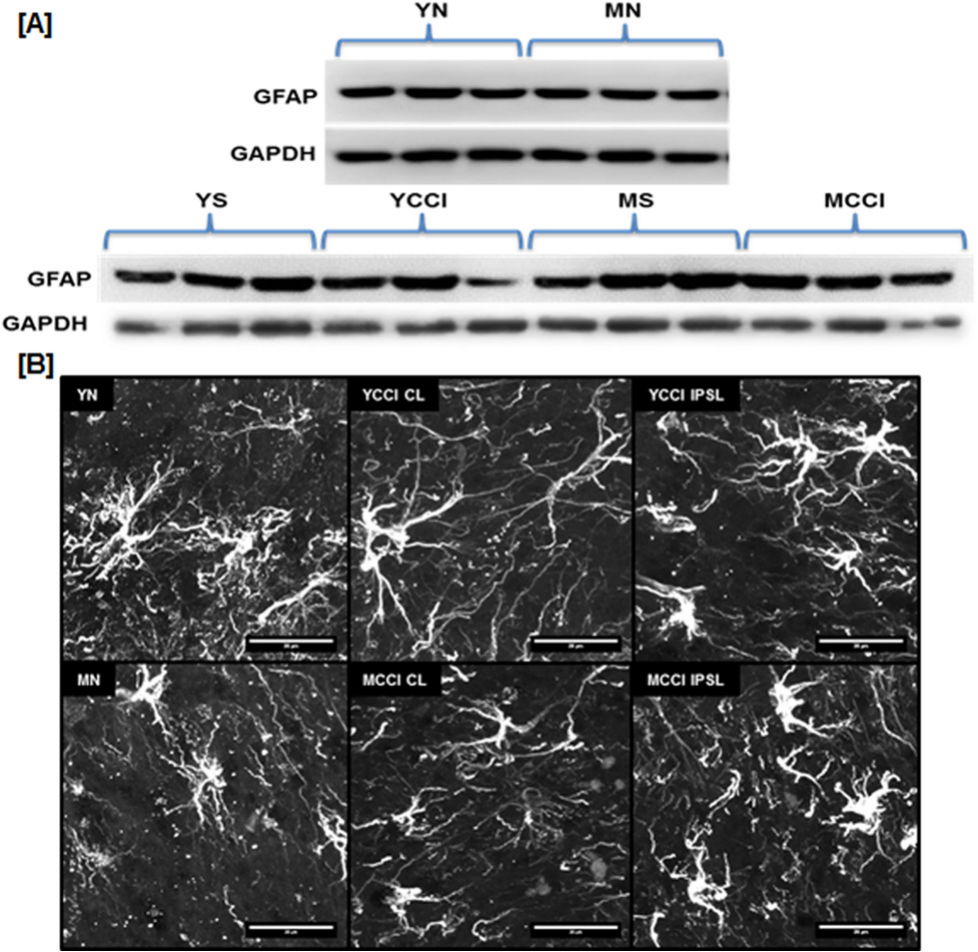
GFAP expression in lumbar spinal cords from young and middle-aged naïve (YN and MN) or post-CCI day 7 animals (YCCI and MCCI). (A) Immunoblots of lumbar spinal cord extracts taken from naïve hemisections or 7 days post surgery that were probed with antibody against GFAP and loading control GAPDH (n = 3 per group). (B) GFAP immunofluorescence of dorsal horn astrocytes. Lumbar spinal cords were obtained from young and middle-aged naïve (YN and MN) or post-CCI day 7 animals (YCCI and MCCI), either ipsilateral (IPSL) or contralateral (CL) to injury. Optical sections (0.75 μm optical sections through 25 μm thick specimens) were acquired with a LSCM and 60x 1.4 NA objective lens, and the image stacks were then recombined and rendered in 3D. Scale bar = 20 μm

Immunoblotting results (Fig 7A) showed no age-related differences in GFAP protein expression between naïve LSC samples nor any age or injury induced differences between IPSL LSC samples from CCI animals compared to sham controls. Astrocytes of the IPSL dorsal horns of both young and middle-aged CCI animals were visualized with GFAP antibody. Injury induced the appearance of a hypertrophic, ramified morphology (Fig 7B right), typical of astrocytes following nerve injury [53, 54]. No significant age-related morphologic differences were observed in contralateral sections from injured animals, nor were any age-induced changes observed in dorsal horn sections from naïve animals (Fig 7B; and S1 Fig). The absence of injury-induced GFAP expression is consistent with several previous studies of CCI of young adults up to post-injury day 14 [55, 56], though another group reported increased expression by post-CCI day 7 [57]. Our data suggest that dorsal horn microglia, but not astrocytes, undergo substantial morphologic/phenotypic changes by middle age, and that GFAP protein expression in astrocytes does not necessarily accompany the establishment of pain following peripheral nerve injury.

#### IV. GFAP Protein Expression in Lumbar DRG

**Fig 8:**
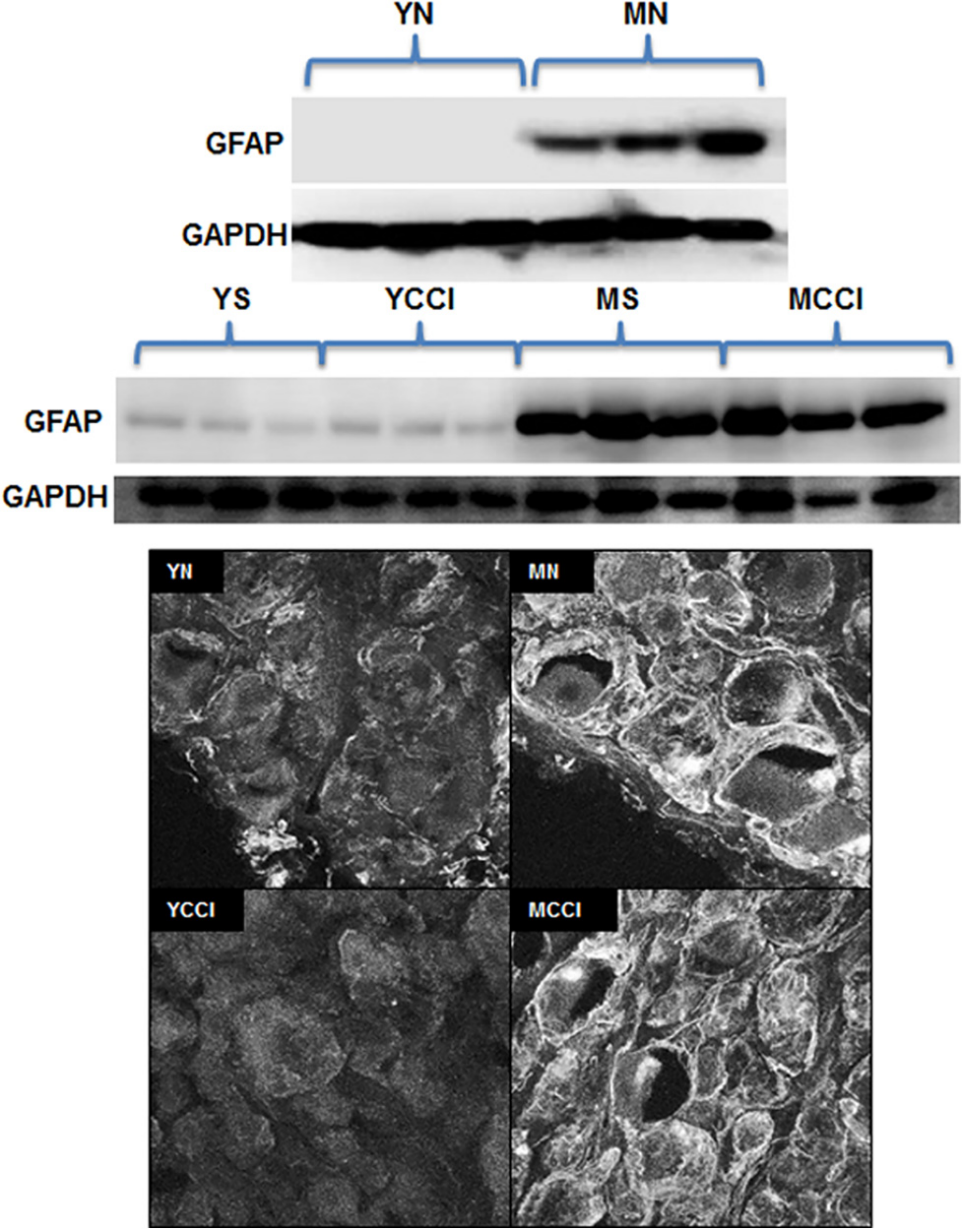
GFAP protein expression in young and middle-aged sham or CCI or naïve DRG. (A) Immunoblots of DRG extracts were probed with antibody against GFAP or loading control GAPDH antibody. Upper panel: young and middle-aged naïve (YN and MN) DRG; lower panel: young and middle-aged sham (YS and MS) or CCI (YCCI and MCCI) DRG. (B) Typical immunofluorescence confocal images of 25 μm thick sections of DRG stained with GFAP antibody. Confocal images were acquired with a LSCM and 60 X 1.4 NA objective lens. L4 and L5 DRG were obtained ipsilateral to injury. Scale bar = 50 μm

GFAP is mainly expressed in satellite glia surrounding primary sensory neurons, although its expression has been reported only under pathologic conditions, such as nerve injury [58–61]. Our immunoblot results (Fig 8A Bottom) showed no significant increase in GFAP protein levels in DRG from CCI animals at day 7, yet levels were markedly elevated in DRG from older sham and CCI animals. These increases were entirely a function of age, as similar elevations were observed in the older naïve adults (Fig 8A Top). It should be noted that the marked effect of age on GFAP protein levels contrasts with the unchanged levels of GFAP mRNA. A prior study had also found discordance between levels of GFAP mRNA and GFAP protein in young adult rats [49].

Images of DRG sections stained with GFAP antibody (Fig 8 B) showed enhanced immunofluorescence intensity in cells ringing the neurons in middle-aged DRG (naïve or CCI) compared to those from young DRG (naïve or CCI), and are consistent with identification of these cells as satellite glia, the principal source of GFAP expression in DRG [58]. While the age related changes are substantial, we did not observe any CCI induced increases in GFAP expression or its localization in DRG. This contrasts with a prior study of young adult rats subjected to CCI that showed increased GFAP immunoreactivity in satellite glia one week following CCI [52]. Whether strain differences or degrees of nerve injury might explain the discrepancy between our results and theirs is unclear.

### Thermal and Mechanical Hyperalgesia and Cold Allodynia following CCI

**Fig 9:**
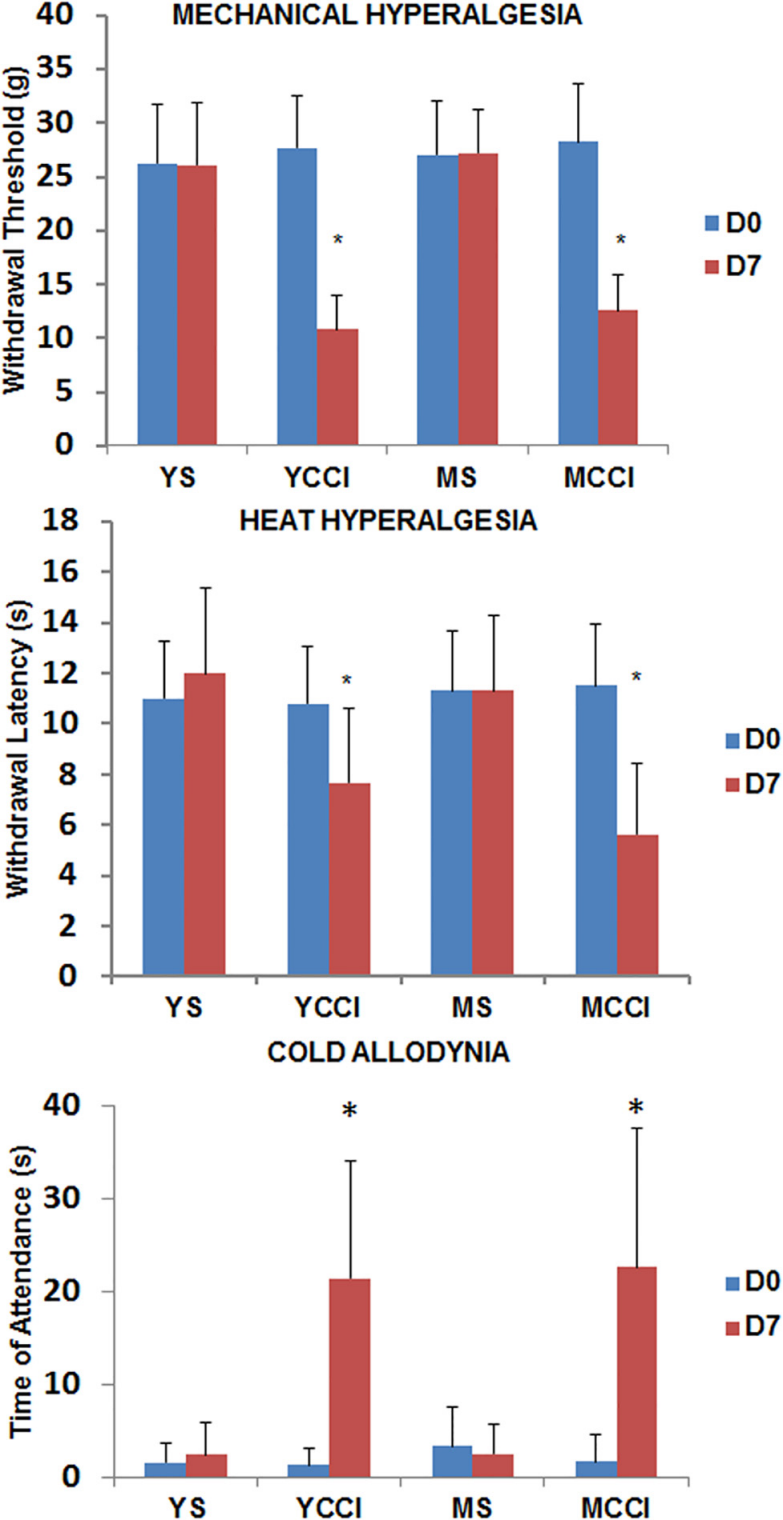
Evoked pain responses at post-injury day 7 in young and middle aged rats before or after sham or CCI surgery. Mechanical hyperalgesia (Top), measured as threshold pressure eliciting paw withdrawal; heat hyperalgesia (Middle), measured as latency time to hind paw withdrawal from a radiant heat source; cold allodynia (Bottom), measured as time of attendance to the affected paw. Results are presented as means +/- SD, n = 11 YS, YCCI, MCCI and n = 9 MS. Data were subjected to Two-Way ANOVA comparing day 7 to pre-injury baseline and differences due to age. * p< 0.0001 comparing day 7 to baseline; there were no significant differences related to age. Similar results were obtained on Day 3, and on Day 7 comparing ipsilateral to contralateral hind paw responses (S2 Fig).

Behavioral measurements were conducted just prior to surgery (day zero), and post injury day 3, and 7 day (Fig 9). Compared to age-matched sham controls, all CCI animals had substantially reduced paw withdrawal latencies in response to radiant heat (only IPSL to injury), reduced thresholds of paw lifting in response to static mechanical pressure, and increased attention times to the paw following a brief cold stimulus (acetone evaporation). No discernable differences were observed between 3 and 17 month-old rats at baseline or 7 days post-CCI across the three response modalities (Fig 9). Similar results were obtained on day 3, and comparing the IPSL to responses obtained on the CL side (S2 Fig). There were also no significant age-related differences at baseline (Fig 9).

### Summary of Results

Expression of mRNA markers, Iba1, and GFAP, and microglial morphologies are summarized in Table 7 (spinal cord), and Table 8 (DRG). The results are presented as comparisons across age and injury. Our data demonstrate substantial age-related differences in expression of numerous mRNA markers, in Iba1 levels and in microglial morphology. Distinctions following CCI in the lumbar spinal cord, however, were subtle or absent. In DRG, the principal age-associated differences were the blunted neuroinflammatory responses following injury and constitutive expression of GFAP localized to satellite glia.

**Table 7:** Summary of multiplex assay results and immunofluorescence imaging of the LSC.

Comparison	Neuroinflammatory mRNA Markers	Iba1 Protein Levels	Density Microglia	P2/P1 Microglia
**MN/YN**	+ATF3, +CCL2, +CD2, +CD3E, +CD45, +CD68, +IL6, +TNFα, +TGFβ1	+++	~	+++
**MS/YS**	+CD2, +CD3E, +CD45, +CD68, +TGFβ1	+++	~	~
**MCCI/YCCI**	-ATF3,-CCL2, +CD2, +CD3E, +CD68	~	~	~
**YCCI/YS**	+++ATF3, +++CCL2, +CD45, +++IL6	+++	+++	+++
**MCCI/MS**	++ATF3, +CCL2, +CD68, +IL6	~	+++	~

LSC = lumbar spinal cord; MN = middle-aged naïve; YN = young naïve; MS = middle-aged sham; YS = young sham; MCCI = middle-aged CCI; YCCI = young CCI. + sign indicates ratios significantly > 1 but < 2-fold; ++ > 2-fold but < 3; +++ > 3-fold;–sign indicates ratios significantly < 1; and ~ sign indicates ratio ~ 1. Density of microglia refers to the dorsal horn, and for CCI, the ipsilateral side 7 days post-injury.

**Table 8:** Summary of multiplex assay results and immunofluorescence imaging of the DRG.

Comparison	Neuroinflammatory mRNA Markers	GFAP Protein Levels	GFAP + Satellite Glia
**MN/YN**	+ATF3	+++	MN only
**MS/YS**	CD45, TGFβ1 (both trending +)	+++	MS only
**MCCI/YCCI**	-CD68,-IL6	+++	MCCI only
**YCCI/YS**	+++ATF3, +CCL2, ++CD2, +CD45, ++CD68, ++GFAP, ++IFNG, +++IL17, ++IL1β, +++IL6, +++TNFα	~ low	~ ND
**MCCI/MS**	+CCL2, +++IL6	~ high	~ detected

LSC = lumbar spinal cord; MN = middle-aged naïve; YN = young naïve; MS = middle-aged sham; YS = young sham; MCCI = middle-aged CCI; YCCI = young CCI. + sign indicates ratio significantly > 1 but < 2-fold; ++ > 2-fold but < 3 fold; +++ > 3-fold;–sign indicates ratios significantly < 1; and ~ sign indicates ratio ~ 1. ND = not detected.

## Discussion

In our study, age alone increased expression of cytokine, stress, T cell and microglial markers in LSC from sham operated and naïve middle-aged adults. In a previous study of aging spinal cord, IL1β, Iba1, and IFNγ protein levels were increased in healthy older adults [17]. Similarly, IL1β, TNFα, IL6, and TGFβ1 mRNA concentrations were also elevated in various regions of the aging rat, mouse and primate brain [5–7], with microglia being a major source in the rodent brain [62, 63]. Our results generally agree, and suggest important roles for T-cells and microglia in establishing the neuroimmune status of the healthy middle-aged lumbar spinal cord.

Differences in dorsal horn microglial morphologies between young and middle-aged animals were striking. We conclude that most dorsal horn microglia in the healthy middle-aged LSC acquire morphologies that are atypical in younger adult spinal cord. Previous studies by Stuesse, Kullberg and others [14, 15] have reported changes in microglial morphologies in spinal cords of senescent rats, as well as increased expression of activation markers, including Iba1, CD68 and CD11b. Increased numbers of microglia with comparable “activated” morphologies were also reported for aging canine [64] and rat [14] spinal cord, facial nucleus [65], as well as in various regions of the rodent brain [7], and in human brain samples, though process beading and various cytoplasmic changes were also reported in the latter [66].

A recent analysis of brain microglia isolated from young and senescent adult mice, supports the idea that microglia develop unique phenotypes with age, and has helped define the senescent microglial transcriptome in brain [63]. Brain microglial senescence is associated with up-modulation of most alternative-priming genes, down regulation of TGFβ1, and unchanged levels of IL10 mRNA. Nearly half of classical priming markers were up modulated with age, including TNF. This transcriptomic profile suggests that aging microglia may have contributed to many of the expression changes we observed in whole spinal cord from healthy middle-aged adults.

In our study, satellite glia, which play a critical role in the inflammatory responses to nerve injury [58, 60], expressed high levels of GFAP in middle age, while the sensory neurons they surround, expressed the stress marker, ATF3. These new results raise questions about the functioning of satellite and microglia in older healthy adults, their interactions with nearby neurons, and whether these age-related changes influence their response to injury or infection, in particular the establishment and resolution of neuroinflammation.

We found that spinal cord inflammatory responses to CCI were similar in young and middle-aged animals at a time when heat, cold, and mechanically evoked pain responses were fully established in both age groups. In young and middle-aged spinal cord, CCI also induced similar changes in dorsal horn astroglial and microglial morphologies. On the other hand, DRG from older animals showed a surprisingly blunted neuroimmune response following CCI that did not correlate with any differential response in pain behaviors. Taken together, our results support the conclusion that a para-inflammatory state develops in the LSC and DRG by middle age. Nonetheless, emergence of this state did not influence the establishment of neuropathic pain. Previous studies have reported substantial delays in recovery from mechanical hyperalgesia following nerve injury (CCI) in senescent rats compared to young adults [24, 25]. Although not directly addressed in humans, epidemiologic studies also suggest delayed resolution and increased pain persistence, plateauing in middle age [37, 38]. It is possible that the age-related para-inflammatory state we describe may be more relevant to persistence than to establishment of neuropathic pain.

Most previous studies of aging and nociceptive responses focused on older rodent models (rats or mice > 20 months of age). Some reported enhanced heat sensitivities in senescent rats [67, 68], but no significant age-related differences in mechanical hyperalgesia [21] following nerve injury; whereas, others reported diminished sensitivities in senescent compared to middle-aged and young adults subjected to nerve injury [64, 69]. Increased heat and cold sensitivity, measured by operant behaviors, have been reported in healthy aging rats and aging animals with acute inflammatory pain [70], though no differences in evoked nociceptive responses were observed. Interestingly, neuropathy-induced depression and loss of cognitive function are worsened in older animals, particularly in middle age [71], supporting the idea that age-related emotional and cognitive components influence perceived pain level and duration, an idea of relevancy to chronic pain patients.

Many of the neuroimmune responses in young CCI animals that we observed closely track those of previous studies that found increased expression of ATF3, CCL2, IL6, IL1β, and microglial activation marker CD11b in the LSC [49, 55]. As others had reported, we found no significant change in IL10 [50, 72] or TNFα mRNA levels [51, 72] 7 days post-CCI. Surprisingly, we observed no significant increases in T cell markers (with exception of CD45) in young or middle-aged LSC following CCI. CD45, a general leukocyte marker, is known to increase following nerve injury, but the bulk of the injury-induced expression has been attributed to activated microglia or invading macrophages [73], and not to invading T-cells. Previous work had shown T cell invasion following CCI [40, 52] and other types of neuropathic injury [33, 74, 75]. Results from numerous studies, using multiple independent approaches have found an important role for T-cell invasion in development and maintenance of neuropathic pain [76]. We did not detect increases in T cell markers following CCI in young LSC, suggesting that our method of causing injury is not as severe as that of other investigators who demonstrated T cell invasion following CCI

Elevation of T cell markers in the sham and naïve middle-aged LSC suggest invasion of the spinal cord parenchyma by these immune cells in the absence of nerve injury. Earlier studies of aging brain had shown increased numbers of CD3+ T-cells in.the white and gray matter of numerous brain regions in middle-aged and senescent animals, that were undetected in young adult tissue except within the choroid plexus and meninges [77]. Since our markers were also of a general type, the functional phenotypes, e.g. Th1, Th2 or Treg, in the middle age spinal cord remain to be determined. The lack of age-related changes in IL17a transcripts, however, suggests that these T-cells do not fall into the Th17 class [78]. How T cells gain entrance to the parenchyma is unknown, but signals from aging glia could serve as chemoattractants [79], perhaps facilitated by increased permeability of the microvasculature [80].

Inflammatory responses in the aging spinal cord and DRG following peripheral nerve injury differed from reports of exaggerated reactions to infection or injury of the aging brain. For example, neuroimmune responses to LPS injection or peripheral infection [8, 81–84] or to brain trauma [85, 86] were amplified in aging rat and mouse. Enhanced microglial proliferation has been reported following facial nerve axotomy [65], as well. On the other hand, studies have reported blunted neuroinflammatory responses in older animals, including: reductions in TNF-α, IL-1β, IL-6, CCL2, CCL5, RANTES and TGFβ1 following stroke [65, 87]; curtailed expression of equivalent markers in middle aged and senescent hippocampus following radiation [88]; diminished levels of arginase, IL-1β, and CCL2 expression in spinal cord after injury [89]; and reduced GFAP [16] and CD11b immunoreactivities [14] in senescent and middle-aged spinal cord following CCI. This general discordance suggests that CNS location and type of insult affect how age/injury interactions influence neuroimmune responses. Our data fit this view.

Unlike the marked increases in levels of neuroimmune markers of the spinal cord and DRG of young adults following nerve injury, DRG neuroimmune markers in older animals showed a blunted or absent response. This was somewhat surprising given that neuropathic pain was fully established when the tissues were harvested. Others have also found marked immune related responses in the DRG following nerve injury and have suggested that these changes are critical to the evolution of neuropathic pain [49–51, 90]. Our results in older animals suggest other possibilities. For example, it is plausible that the inflammatory response peaked earlier in the affected DRG of older animals or that chronic stress in the DRG neurons induced by age alone blunted any changes in marker expression. Comparisons of naïve young and middle-aged DRG showed increased expression of ATF3 in older animals, suggesting stress in aging sensory neurons. Moreover, GFAP protein levels, reflecting satellite glial activation [58, 60], were elevated in the DRG of middle-aged adults, but did not increase following CCI in either age group. Because young and middle age rats developed similar pain sensitivities following CCI, these results suggest that pain development following peripheral nerve injury can be independent of the degree of satellite glial activation or the extent of neuroinflammatory response in the DRG. Thus, evidence of chronic stress in aging primary sensory neurons, coupled with activation of their surrounding support cells in healthy animals could be related to the blunted CCI-induced response in middle age DRG. Further work is needed to explore the implications of these results, and to determine how age-induced changes in primary sensory neurons and satellite glia in DRG, and T-cells and microglia in the LSC, influence other aspects of neuropathic pain, such as pain persistence.

## Supporting Information

S1 Fig Representative GFAP immunofluorescence images of the lumbar spinal cord dorsal horns from young (YN) and middle-aged (MN) naïve animals and 7 days post-CCI. Images were obtained with a LSCM and a 40X 1.4 NA objective lens. We observed no age-related significant differences in astrocyte morphology. Scale bar = 50 μm.
(TIF)

S2 Fig (A) Evoked pain responses post-CCI day 7 in young and middle-aged rats: comparison of ipsilateral versus contralateral responses. CCI injury elicits sensitivity 7 days post surgery in each modality (N = 11 per group) in the paw that is ipsilateral (IPSL) to injury, but not the contralateral (CL) paw. Two Way ANOVA * p ≤0.0001 shows significance of differences between ipsilateral and contralateral paw. There were no significant differences related to age. (B) Evoked pain responses post-CCI in young and middle-aged rats: Day 3 Post CCI Mechanical, Heat, and Cold responses. CCI injury elicits sensitivity 3 days post surgery in each modality in comparison to age-matched sham controls (N = 6 per group). Two Way ANOVA multiple * p ≤0.0001 of each condition compared sham controls. No significant age-related differences were observed.
(TIF)

S1 File 3D Rotating Image of Young Dorsal Horn Microglia. These combined Z stack images, which provide a detailed view of Iba1+ microglia, typify the morphologies seen in the young lumbar spinal cords.
(GIF)

S2 File 3D Rotating Image of Middle-Aged Dorsal Horn Microglia. These combined Z stack images, which provide a detailed view of Iba1+ microglia, typify the morphologies seen in the middle-aged lumbar spinal cords.
(GIF)

S3 File 3D Rotating Image of Young CL CCI Dorsal Horn Microglia. These combined Z stack images, which provide a detailed view of Iba1+ microglia, typify the morphologies seen in the middle-aged lumbar spinal cords.
(GIF)

S4 File 3D Rotating Image of Young IPSL CCI Dorsal Horn Microglia. These combined Z stack images, which provide a detailed view of Iba1+ microglia, typify the morphologies seen in the middle-aged lumbar spinal cords.
(GIF)

S5 File 3D Rotating Image of Middle-aged CL CCI Dorsal Horn Microglia. These combined Z stack images, which provide a detailed view of Iba1+ microglia, typify the morphologies seen in the middle-aged lumbar spinal cords.
(GIF)

S6 File 3D Rotating Image of Middle-aged IPSL CCI Dorsal Horn Microglia. These combined Z stack images, which provide a detailed view of Iba1+ microglia, typify the morphologies seen in the middle-aged lumbar spinal cords.
(GIF)
