# Supplementary figures and images for: Neuroimmune and Neuropathic Responses of Spinal Cord and Dorsal Root Ganglia in Middle Age
Source: PLoS One. 2015 Aug 4;10(8):e0134394. doi: 10.1371/journal.pone.0134394 (PMC4524632; doi:10.1371/journal.pone.0134394)

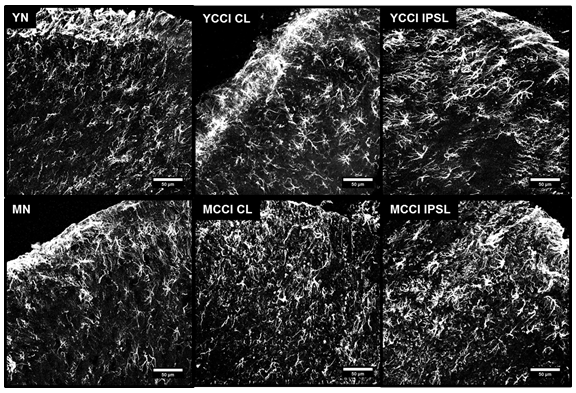

Supplement: S1 Fig — Images were obtained with a LSCM and a 40X 1.4 NA objective lens. We observed no age-related significant differences in astrocyte morphology. Scale bar = 50 μm. (TIF) [file pone.0134394.s001.tif]

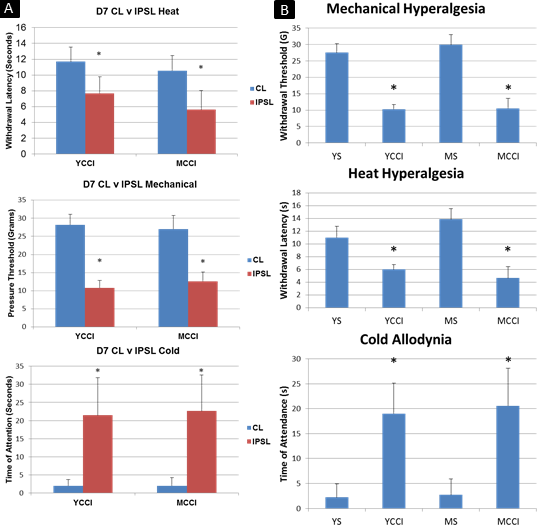

Supplement: S2 Fig — CCI injury elicits sensitivity 7 days post surgery in each modality (N = 11 per group) in the paw that is ipsilateral (IPSL) to injury, but not the contralateral (CL) paw. Two Way ANOVA * p ≤0.0001 shows significance of differences between ipsilateral and contralateral paw. There were no significant differences related to age. (B) Evoked pain responses post-CCI in young and middle-aged rats: Day 3 Post CCI Mechanical, Heat, and Cold responses. CCI injury elicits sensitivity 3 days post surgery in each modality in comparison to age-matched sham controls (N = 6 per group). Two Way ANOVA multiple * p ≤0.0001 of each condition compared sham controls. No significant age-related differences were observed. (TIF) [file pone.0134394.s002.tif]

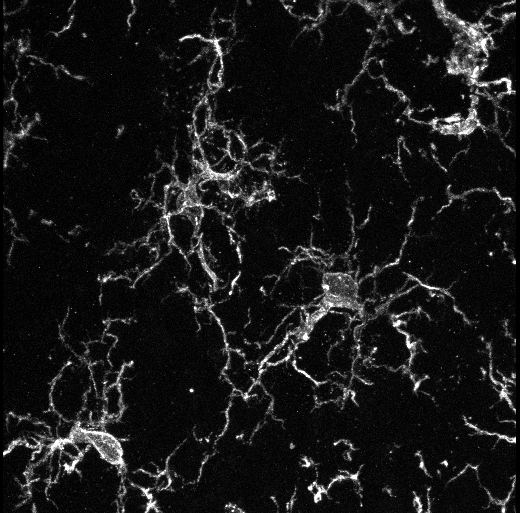

Supplement: S1 File — These combined Z stack images, which provide a detailed view of Iba1+ microglia, typify the morphologies seen in the young lumbar spinal cords. (GIF) [file pone.0134394.s003.gif]

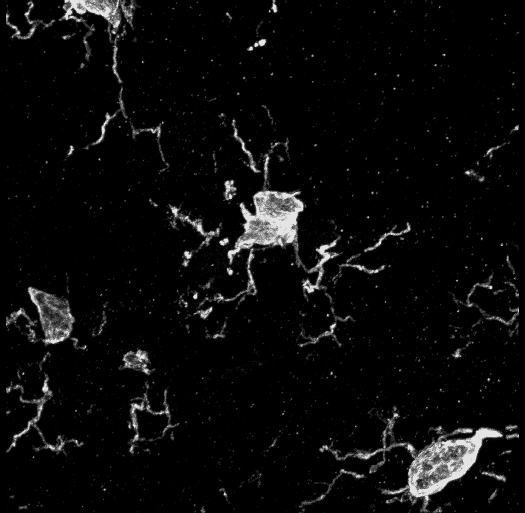

Supplement: S2 File — These combined Z stack images, which provide a detailed view of Iba1+ microglia, typify the morphologies seen in the middle-aged lumbar spinal cords. (GIF) [file pone.0134394.s004.gif]

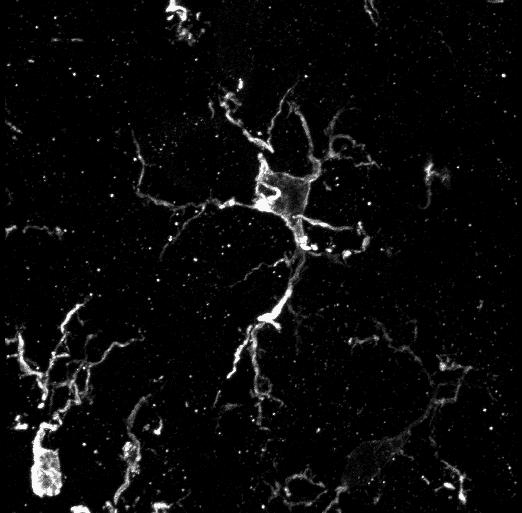

Supplement: S3 File — These combined Z stack images, which provide a detailed view of Iba1+ microglia, typify the morphologies seen in the middle-aged lumbar spinal cords. (GIF) [file pone.0134394.s005.gif]

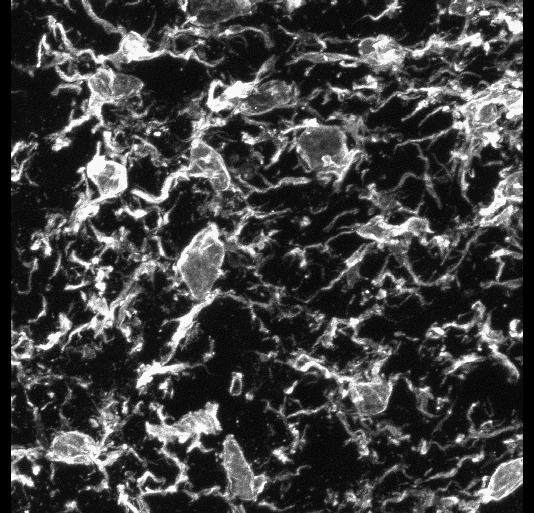

Supplement: S4 File — These combined Z stack images, which provide a detailed view of Iba1+ microglia, typify the morphologies seen in the middle-aged lumbar spinal cords. (GIF) [file pone.0134394.s006.gif]

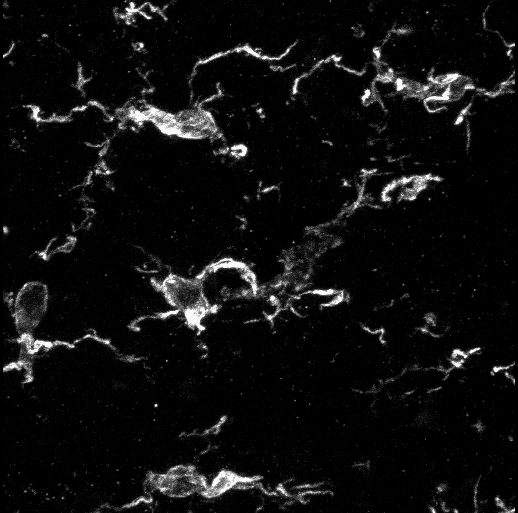

Supplement: S5 File — These combined Z stack images, which provide a detailed view of Iba1+ microglia, typify the morphologies seen in the middle-aged lumbar spinal cords. (GIF) [file pone.0134394.s007.gif]

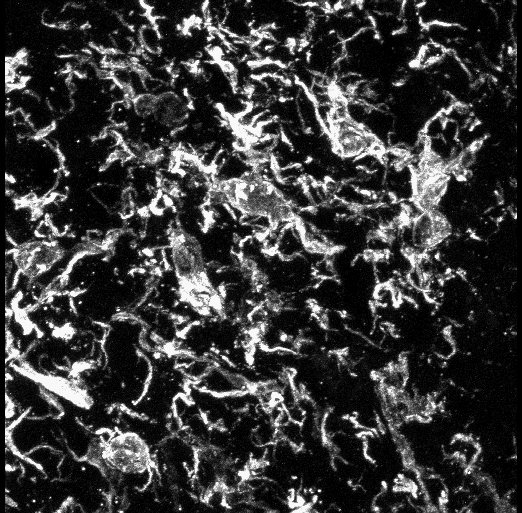

Supplement: S6 File — These combined Z stack images, which provide a detailed view of Iba1+ microglia, typify the morphologies seen in the middle-aged lumbar spinal cords. (GIF) [file pone.0134394.s008.gif]
